# Muscle ultrasound shear wave elastography for detection of early onset lower limb ischemia in patients with veno-arterial extracorporeal membrane oxygenation

**DOI:** 10.1186/s40635-023-00576-6

**Published:** 2023-12-11

**Authors:** Mariya Maslarska, Sven Piepenburg, Dirk Westermann, Alexander Supady, Christoph Hehrlein

**Affiliations:** 1 Department of Cardiology and Angiology, Medical Center - University of Freiburg, Hugstetterstr. 55, 79106 Freiburg im Breisgau, Germany; 2 Faculty of Medicine, University of Freiburg, Freiburg im Breisgau, Germany; 3 Interdisciplinary Medical Intensive Care, Medical Center - University of Freiburg, Freiburg im Breisgau, Germany

**Keywords:** Critical limb ischemia, Intraarterial cannula, Shear wave elastrography ultrasound, VA-ECMO

## Abstract

**Background and objectives:**

Limb ischemia or compartment syndrome, requiring surgery, are some of the frequent cannula-related complications in patients supported with veno-arterial extracorporeal membrane oxygenation (VA-ECMO). The purpose of this exploratory study is to depict and evaluate the dynamic changes in the lower limb muscles with ultrasound shear wave elastography as marker for early lower limb ischemia.

**Methods:**

Eleven patients with VA-ECMO after cardiac arrest were included in this study. Seven patients received distal perfusion cannula (DPC) after implantation of the VA-ECMO, whereas 4 had no DPC after VA-ECMO. Compartment syndrome was clinically excluded in all patients. Both lower limbs, e.g., with and without arterial cannula, were monitored with near-infrared spectroscopy (NIRS) for the oxygen saturation of the local tissue. We performed ultrasound shear wave elastrography (SWE) to assess dynamic changes of the medial gastrocnemius muscle at maximum passive muscle stretch (exercise) of both legs. Color-coded duplexsonography was conducted to examine the blood flow velocity of the popliteal artery of the lower limb.

**Results:**

We found no difference between DPC and no DPC (*p* = 0.115) during use of VA-ECMO. However, we detected marked lower limb muscle perfusion deficits of cannulated (58.9 ± 13.5 kPa) vs. cannula-free limb (95.7 ± 27.9 kPa: *p* < 0.001), applying SWE. No relationship was detected between NIRS measurements and SWE values (kPa) of both lower limbs. The mean peak systolic velocity of the popliteal artery at the cannulated side (30.0 ± 11.7 cm/s) was reduced compared to the non-cannulated side (39.3 ± 18.6 cm/s; *p* = 0.054).

**Conclusions:**

Regardless of DPC after implantation of VA-ECMO, the gastrocnemius muscles seem to lose function due to cannula-related microcirculatory deficits. Muscle function analysis via SWE combined with NIRS might offer a sensitive indicator for early onset leg ischemia during VA-ECMO-related arterial cannulation.

## Background

Patients after cardiac arrest supported with veno-arterial extracorporeal membrane oxygenation (VA-ECMO) may suffer from multiple complications. Limb ischemia is among the most severe and frequent complications with an incidence rate ranging between 10 and 50% during VA-ECMO [1–3]. Close to 12% of the patients, who develop ischemia, require surgical fasciotomy due to compartment syndrome [3]. The most prominent risk factors for limb ischemia include the size of the arterial cannula and the arterial vessel diameter as well as patient age and female sex [4–6]. Larger arterial cannulas are linked to ischemic events of the limbs due to possible flow obstruction [5]. In this context, the ratio between arterial diameter and the cannula plays a major role [7]. The vessel diameter and patient age correlate directly with each other, since arteries increase in size with age. Furthermore, male sex is associated with larger arterial vessels [8]. Peripheral artery disease increases further the risk for limb ischemia in patients on VA-ECMO [4].

Strategies for prevention of vascular complications include the insertion of a distal perfusion cannula (DPC) into the superficial femoral artery [6, 9]. In a retrospective study of 91 patients, 55 received a percutaneous DPC prophylactically and did not develop limb ischemia during the further treatment [9]. However, despite the optimization of lower limb blood flow with DPC, VA-ECMO patients require strict monitoring for early signs of limb ischemia of the cannulated leg because of the increased risk of thromboembolism, loss of DPC function or possible injury of the artery [6, 10]. Current standards for monitoring and early detection of limb ischemia include clinical examination, color-coded duplex ultrasonography and near-infrared spectroscopy (NIRS) [6]. Duplex ultrasonography is a sensitive method to determine the perfusion status of the lower limb arteries (macrocirculation) by determining the peak systolic velocity (PSV) and the degree of vascular stenosis or obstructions. However, the assessment and interpretation of haemodynamic parameters in medium size arterial vessels are complex, especially in patients with VA-ECMO. Arterial duplex sonography provides sufficient monitoring the macrocirculation and larger blood vessels but is no suitable to detect microcirculation disturbances, specifically in cases with non-pulsatile flow [11]. Near-infrared spectroscopy (NIRS) has become an important diagnostic approach for the early detection of limb ischemia in patients with large-bore cannulation for VA-ECMO. NIRS measures skin tissue saturation and oxygenation by applying light with near-infrared wavelengths between 650 and 1100 nm. Light absorbance in the local tissue correlates with the concentration of oxygenated and deoxygenated forms of haemoglobin and thus depicts the regional oxygen saturation (rSO_2_) [6, 12]. Current clinical practice has established values for tissue saturation greater than 60% as marker for sufficient perfusion. In contrast, rSO_2_ lower than 50% for longer than 4 min is a sensitive predictor for lower limb ischemia [13].

Shear wave elastography (SWE) is a novel ultrasound method to explore the mechanical and elastic properties of muscle tissue, which can provide insights into the level of perfusion in the examined area [14]. Shear waves, generated by the ultrasound transducer, spread through different tissues with different velocities, depending on the mechanical properties of the examined region. The tissue stiffness or elasticity (kPa) is defined by the product of shear wave velocity and material density [14, 15]. Experimental studies have determined physiological elasticity values of lower limb muscles, such as the gastrocnemius muscle [16]. Explorative studies have depicted significant differences in the muscle elasticity in a relaxed and contracted state [16, 17]. Our recent study [18] obtained median and cutoff values for muscle elasticity during the active exercise of the medial gastrocnemius muscle of healthy participants and chronic heart failure patients. Healthy individuals achieved a median of 86.3 kPa during active stretching of the gastrocnemius muscle compared to a median of 59.8 kPa in patients with chronic heart failure [18]. As a result, a cutoff of 81.1 kPa was associated with high sensitivity in detecting muscle weakness in the lower limbs in our small study cohort [18]. Although these results were obtained with a limited number of patients, values measured with SWE could act as an indirect marker for possible malperfusion with oxygen in the muscle microcirculation [18, 19]. In this way, SWE could provide additional information for potential lower limb ischemia in patients with VA-ECMO, where other diagnostic options are limited.

In this explorative, first-in-human study we analysed whether shear wave elastography at rest and during passive stretching of the peripheral lower limb muscles is a sensitive method for the detection of early limb ischemia during VA-ECMO with or without DPC.

## Methods

### Patient characteristics

This is a single-centre explorative clinical trial. We recruited and examined eleven patients after cardiac arrest supported with VA-ECMO with or without DPC in the interdisciplinary medical intensive care unit (ICU) at the University Medical Center of Freiburg, Germany. VA-ECMO was implanted bifemoral into the left or right femoral artery as the primary arterial insertion side in all patients at the discretion of the treating VA-ECMO specialist at the bedside, with or without ultrasound guidance. SWE was performed within a routine duplex ultrasound examination of the lower limbs. Informed consent was obtained from a close family member or a legal guardian of the patient, since all patients were unable to give an informed consent on their own. The study protocol was approved by the ethics committee of the University Medical Center of Freiburg (No. 21-1463) and conforms to the Declaration of Helsinki.

The main inclusion criteria included patient age of at least 18 years and bifemoral VA-ECMO. Both patients with or without distal perfusion cannula were included. Patients with a history of peripheral artery disease, confirmed by a CT–angiography or a duplex ultrasound at the time of examination, were excluded. Patients, who recently had a surgical intervention of the lower limbs within the post-resuscitation therapy and presented with limited access to the gastrocnemius muscle as region of interest (ROI), were not included. We examined all patients within the first few days after hospital admission. The earliest timepoint for assessment was day 1 after VA-ECMO implantation.

Basic demographic information including age, sex, main diagnosis and current therapy were recorded for each patient. We documented basic VA-ECMO parameters, such as blood flow and cannula size.

### Diagnostic approach: monitoring with NIRS, shear wave elastography (SWE) and color-coded duplexsonography

All patients received NIRS as monitoring of the skin oxygen saturation on both legs in the calf area. NIRS measurements were recorded via self-adhesive electrodes, using an INVOS-Monitor (Medtronic, Minneapolis, USA). NIRS-values above 50% were considered satisfactory with respect to tissue oxygenation according to the local hospital standards [20]. All NIRS measurements were electronically transferred to the patient data management system. For the purpose of this study we recorded NIRS-values from the lower limbs at the time of SWE examinations. Patients did not present any clinical or diagnostic signs of limb ischemia upon inclusion and SWE examination.

We performed ultrasound shear wave elastography within B-mode images obtained from both the left and the right lower limb to detect muscle elasticity changes during passive muscle stretching. We assigned the medial head of the gastrocnemius muscle as the region of interest (ROI). Based on previous SWE protocols, the ultrasound transducer was positioned in a longitudinal direction along the gastrocnemius muscle without additional pressure to the skin [18, 19]. At first, we performed three measurements at rest on both legs. This was followed by an ultrasound examination of passive stretching of the gastrocnemius muscle. Passive stretching was achieved by maximal dorsal flexion of the foot, supported by the same examiner in all patients. We took between three to four SWE measurements followed by a color-coded duplex ultrasonography of the popliteal arteries taking up 5 min of investigational time. No complications at the insertion site of the arterial cannula as well as dislocation of the cannula were observed during passive leg stretching. This form of passive exercise was chosen, since all patients were intubated and sedated and thus unable to perform an active movement by themselves. Furthermore, previous studies have demonstrated a sufficient change in the elasticity values (kPa) during stretching of the gastrocnemius muscle [18, 19]. A passive muscle stretch increases the demand of a microcirulatory blood flow in the muscle and correlates with ischemia-related muscle weakness and with the degree of arterial perfusion in the lower limb [18, 19]. Elevated shear modules of muscles at rest have been suggested to be a reliable early indicator of a compartment syndrome by detecting an increase of the intramuscular pressure [21].

All patients in this study received a color-coded duplex ultrasonography examination of all segments of the popliteal arteries at the cannulated and cannula-free legs. The peak systolic velocity (cm/s) was measured on both sides. SWE and color-coded duplex ultrasound examinations were performed with a standard ultrasound machine (GE Logiq E9, Serial Nr. 135031US3, year 2014, Software Nr. 5536054) using 2–8 MHz ultrasound probes. SWE images were superimposed on a B-Mode image with a color-coded scale, measuring the elastic module in kPa. The variance of kPA-values is demonstrated by the color-coded scale on the left side of the picture, as shown in Fig. 2.

### Statistical analysis

Continuous variables such as SWE measurements at rest and during exercise were documented as means ± standard deviation (SD). We compared these variables between the cannulated and the cannula-free sides, using a paired *T* test. SWE measurements of both legs were compared at resting and exercise states. We used univariate linear regression to establish possible relationship between SWE measurements and NIRS values. Peak systolic velocities of the popliteal arteries between the cannulated and non-cannulated sides were compared with a paired *T* test. Statistical significance was defined below a *p* value of 0.05. IBM SPSS version 28.0 software (Armonk, NY) was used for the statistical analysis.

## Results

### Patient characteristics

All patients included in this study suffered from cardiac arrest caused by myocardial infarction, respiratory failure or rhythm abnormalities. The mean age of all patients (nine male, two female) was 63 years. 8/11 patients (73%) were cannulated with a 17 French arterial cannula; 3/11 patients (27%) had a 15 French arterial cannula. 3/11 patients (27%) were additionally supported with one vasopressor and 7/11 (63%) patients received two different vasopressors, including noradrenaline, adrenaline, vasopressin or dobutamine. One patient did not require any catecholamine therapy. Most of the VA-ECMO patients required a blood flow higher than 2.5 L/min via the extracorporeal life support system (Sorin Centrifugal Pump System or Maquet Cardiohelp). Seven patients obtained DPC. One patient developed lower limb ischemia on the side of the arterial cannula immediately after the VA-ECMO was explanted. This patient had also received DPC during the VA-ECMO support. We observed no cases of lower limb ischemia during the VA-ECMO treatment. 8/11 patients had the venous cannula in the femoral vein on the same side as the arterial cannula. Three patients had the drainage contralateral to the arterial cannula. None of the patients required any further mechanical circulatory support, such as Impella or IABP.

### SWE of gastrocnemius muscle at rest and during passive stretch

**Fig. 1:**
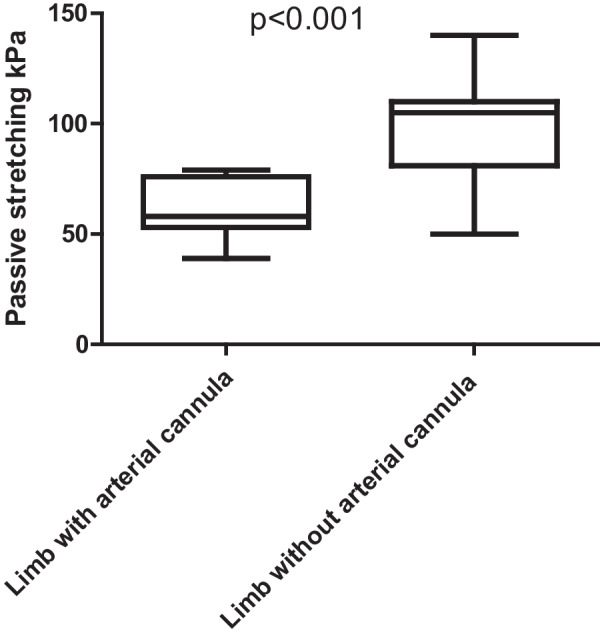
Boxplot graphic for kPa-values during passive muscle stretch between the cannulated and cannula-free sides. Significant difference between the elasticity values on both sides was measured during exercise

On both sides, mean tissue elasticity (kPa) at rest was similar with 10.9 kPa on the cannula side vs. 10.9 kPa on the uncannulated side (*p* = 0.89). During passive stretching of the gastrocnemius muscle we obtained mean elasticity values of 58.9 ± 13.5 kPa for the cannulated side compared to 95.7 ± 27.9 kPa at the non-cannulated side (*p* < 0.001, Fig. 1).

**Fig. 2:**
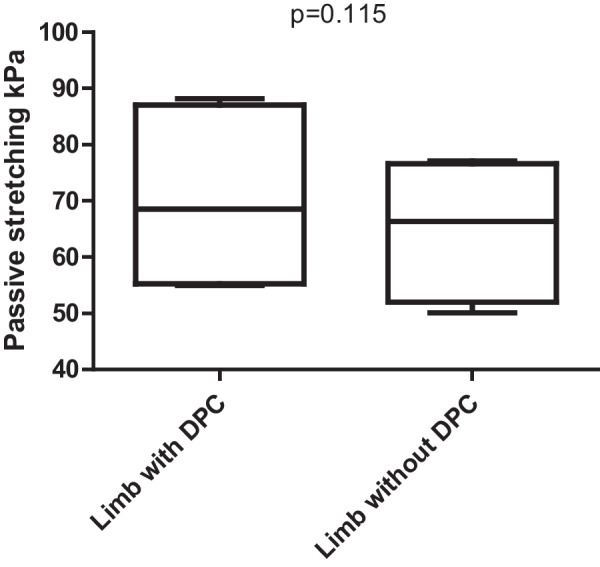
Boxplot graphic of kPa-values of the cannulated side during passive muscle stretching in patients with and without DPC. No significant difference during exercise was observed in patients with or without DPC. No significant difference during passive muscle stretching (exercise)

We compared the mean elasticity results for patients with and without DPC. Patients demonstrated similar elasticity values at the cannulated side during muscle stretching regardless of the presence of DPC (70.4 ± 13.8 kPa with DPC vs. 64.9 ± 13.2 kPa without DPC; *p* = 0.115, Fig. 2).

**Fig.3:**
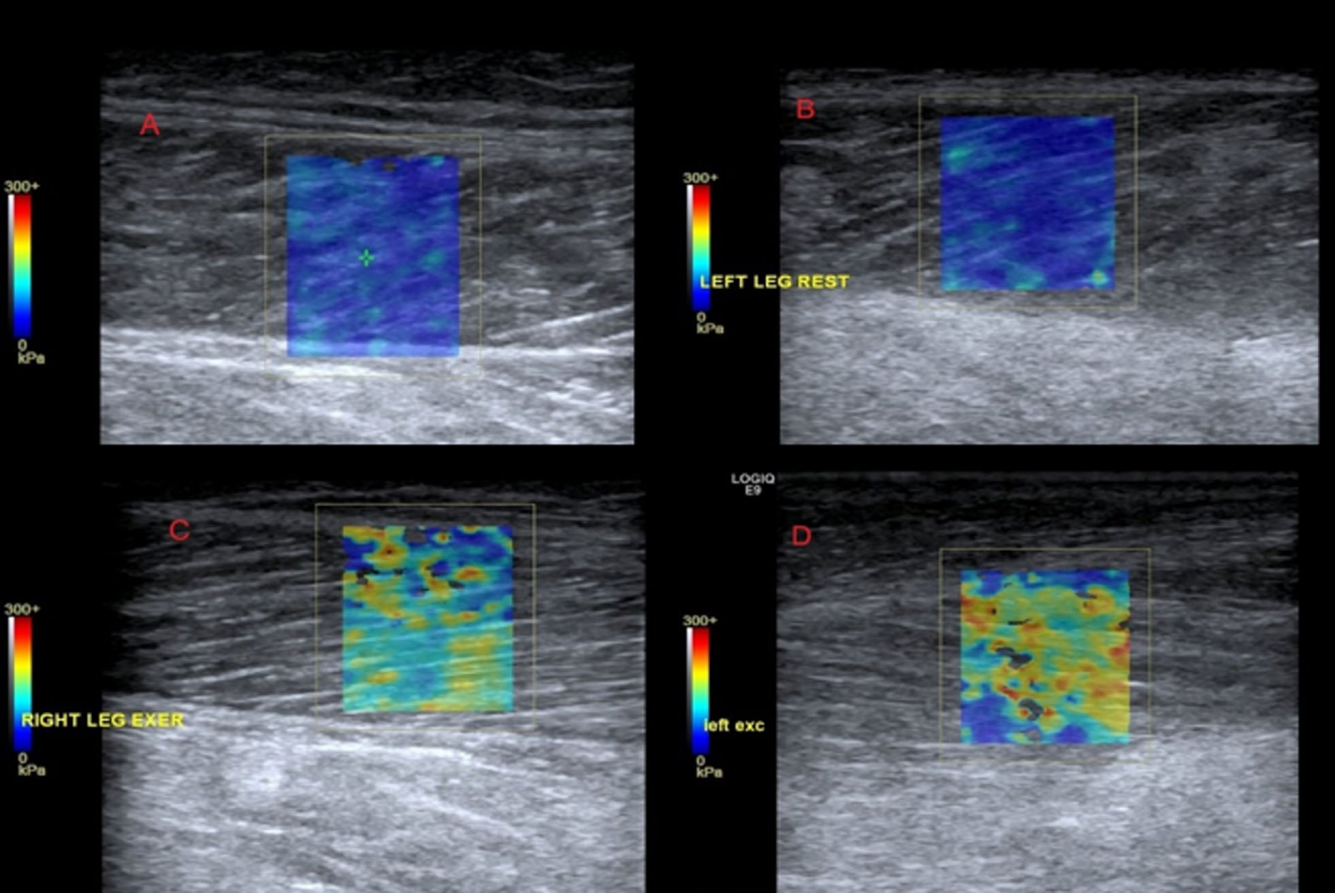
SWE examination of both lower limbs during rest (**A**, **B**) and exercise (**C**, **D**) of both legs: Limb with arterial cannula (**A**, **C**) and limb without arterial cannula (**B**, **D**)

Figure 3 shows an example of SWE images at rest and during muscle stretching of lower limb gastrocnemic muscles. Images A and B depict SWE measurements at rest on the side with the arterial cannula and on the side without cannula, whereas images C and D represent the change in elasticity during passive stretching.

### Relationship between SWE and NIRS measurements

**Fig. 4:**
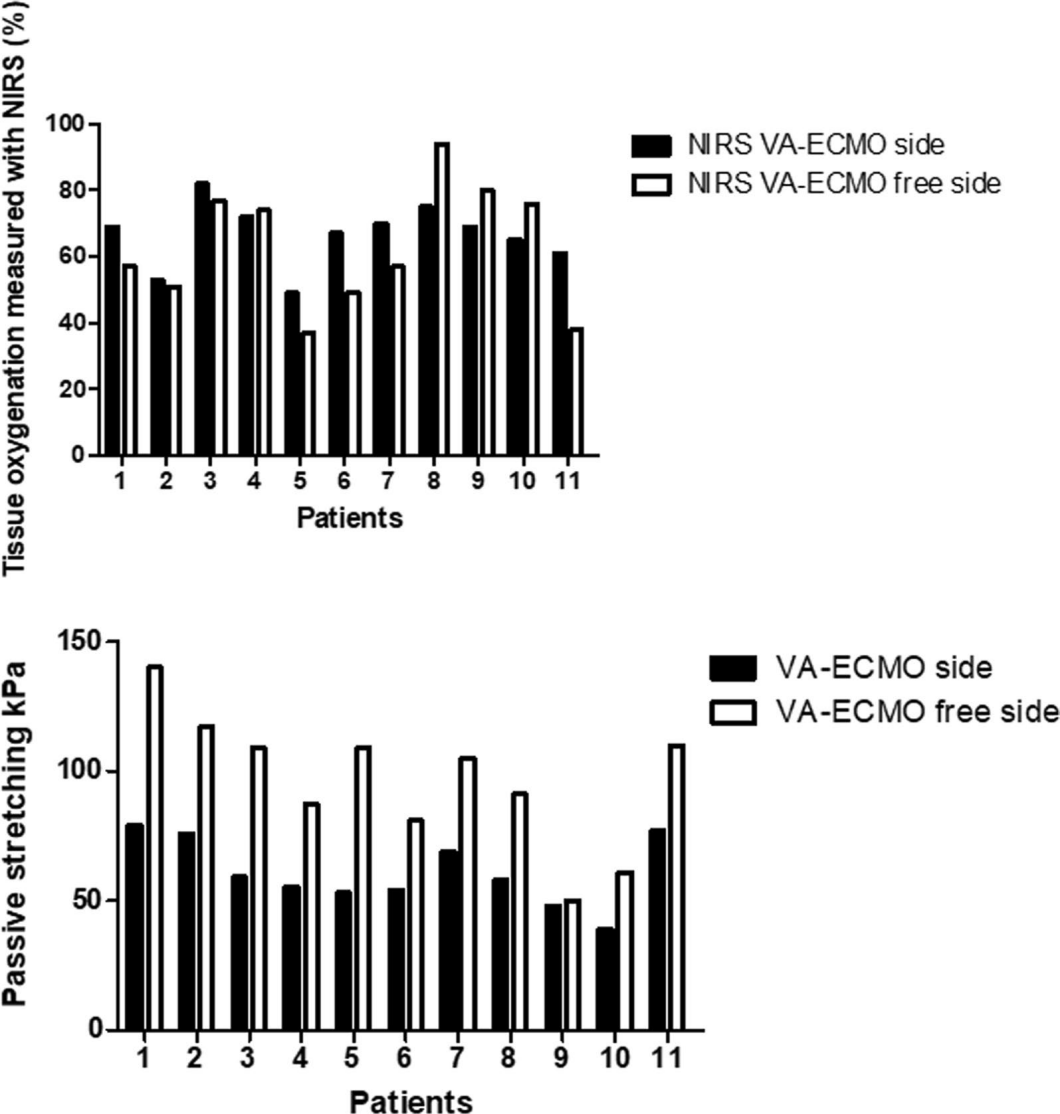
Detection of early onset of lower limb ischemia of cannulated-vs. cannula-free lower limbs of VA-ECMO patients via passive exercise (in both legs) in kPa compared to NIRS. We compared individual kPa-values and NIRS measurements of all patients on both lower limbs

Correlating the kPa stretching values of SWE vs. NIRS as independent variable of the VA-ECMO cannulated leg with a linear regression analysis, we found with an R^2^-coefficient of 0.004 (*p* = 0.79) no relationship between the skin oxygen saturation (NIRS) and muscle perfusion depicted by muscle tissue elasticity. NIRS measurements were not predictive for muscle elasticity during exercise. Figure 4 represents a graphical comparison between individual SWE-values at the cannulated and cannula-free sides compared to the NIRS-measurements of both legs of each patient. NIRS measurements showed no significant variance between the cannulated and cannula-free sides as opposed to SWE values during exercise that varied significantly between the limbs with and without arterial cannulation.

### SWE during passive muscle stretching and systolic peak flow velocity of the popliteal artery

**Fig. 5:**
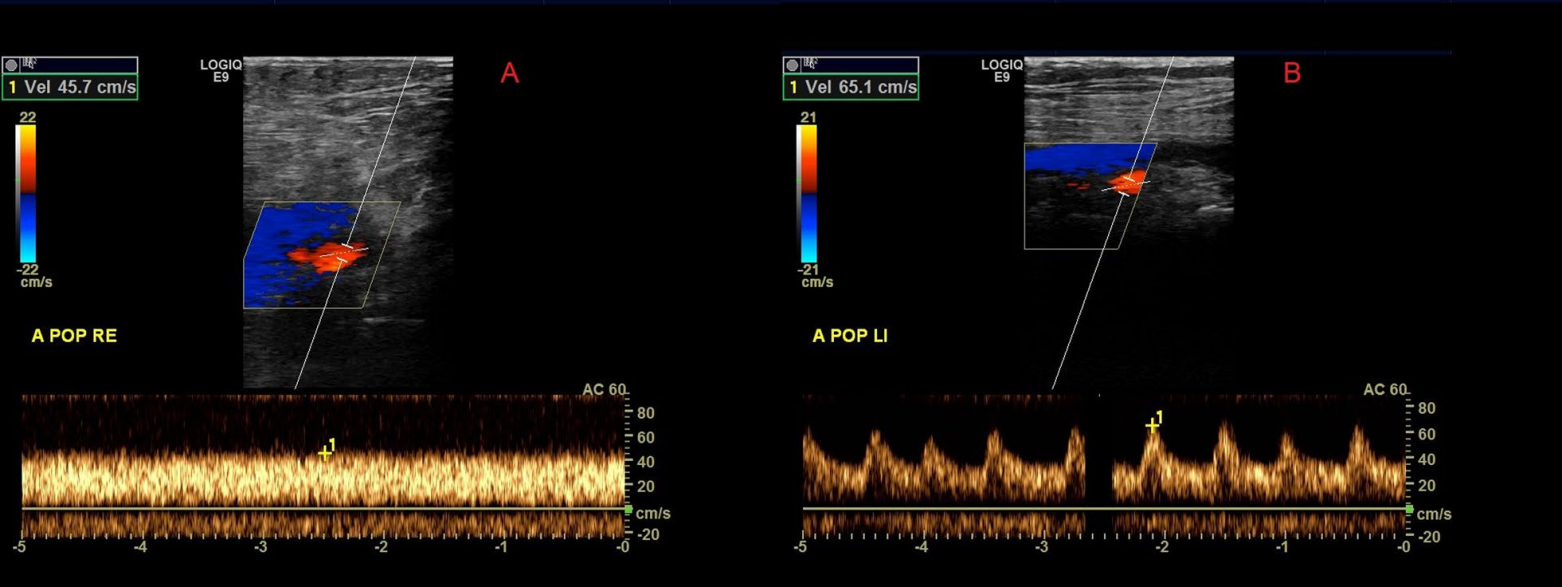
Color-coded duplex ultrasonography images of the leg with an arterial cannula (**A**) and the cannula-free leg (**B**)

We observed pulsatile blood flow in both popliteal arteries despite arterial cannulation in two patients. These patients did not receive DPC. Two patients presented a continuous mean blood flow profile (machine flow) on both limbs. The rest of the patients showed a pulsatile systolic–diastolic blood flow profile with an identifiable systolic peak on the cannula-free leg and continuous blood flow on the cannulated side. Figure 5 depicts an example with the blood flow profile and velocities on both sides. The mean peak systolic velocity on the cannulated side was 30.0 ± 11.7 cm/s was reduced compared to the non-cannulated side (39.3 ± 18.6 cm/s; *p* = 0.054).

## Discussion

We present the results from a first-in-human exploratory study to examine the diagnostic potential of shear wave elastography for the detection of early perfusion deficits in patients with VA-ECMO. So far, diagnostic options for the monitoring of lower limb perfusion and detecting signs of possible ischemia are limited to clinical examination, monitoring of tissue oxygen saturation with NIRS and color-coded duplex ultrasonography [12, 22–24]. Moreover, NIRS skin measurements might be less sensitive for the detection of early onset leg ischemia [20, 25]. We detected similar SWE parameters of the gastrocnemius muscles on both legs (cannulated side and cannula-free side) at rest, regardless of the presence or absence of a DPC. Our findings on rest-kPa values corroborated clinical examination results that no compartment syndrome of the lower limb muscles was present in all studied VA-ECMO patients. These results are consistent with previously published experimental data and confirm that muscle elasticity does not change at rest even at different levels of microcirculation and is probably not influenced by the presence of DPC [18, 19]. We found substantial alterations in muscle function during the stretching, even though exercise was induced in a passive way by one of the examiners and not actively provoked by the patient himself. Piepenburg et al. recently demonstrated a significant kPa-difference during active muscle stretching between the symptomatic and asymptomatic legs of patients with peripheral artery disease (PAD) and media sclerosis [19]. We now detected significantly lower SWE-values on the cannulated side compared to the limb without an arterial cannula in VA-ECMO patients during passive muscle stretching in a similar examination setting [18, 19]. These findings may have further implications for the early detection of muscular ischemia due to microcirculation deficits in patients who are unable to move actively.

### Ultrasound shear wave elastography and NIRS monitoring

The results of this pilot study revealed similar tissue oxygen saturation of the lower limbs on both sides at rest as measured by NIRS despite the arterial cannula, as demonstrated in Fig. 4. We noted NIRS-values higher than 50% at the cannulated side in ten patients. Two patients demonstrated a peripheral saturation lower than 50% at the cannula-free leg. Elasticity results showed significant differences during exercise between both sides. The cannulated leg in all patients exhibited lower muscle elasticity values during passive stretching as a sign of a reduced perfusion compared to the leg without an arterial cannula. These findings suggest that shear wave elastography could offer a sensitive method to detect malperfusion and possible early ischemia in VA-ECMO patients when NIRS monitoring cannot deliver further information. Based on these findings, we hypothesize that shear wave elastography could provide additional clinical information about perfusion deficits at an early stage and assist the diagnostic pathway for lower limb ischemia.

Measurement of skin tissue oxygen saturation offers the possibility to continuously monitor limb and cerebral perfusion. However, NIRS is limited in its relatively low diagnostic accuracy, specifically in cases of hyperbilirubinemia or elevated levels of myoglobin [20, 25]. Vranken et al. examined changes in cerebral and peripheral tissue oximetry in patients with VA-ECMO and different complications such as neurological complications or limb ischemia with the necessity for DPC. Patients with or without neurological complications presented a similar number of desaturation episodes. Tissue oxygen saturation of the non-cannulated lower leg remained constant with a mean of 65% throughout the entire duration of the VA-ECMO therapy. In cases of advanced limb ischemia, where larger peripheral arteries are already involved, NIRS values may or may not drop significantly down to rSO2 of 30% [23]. Compared to NIRS, shear wave elastography provides information about muscular microcirculation and the likelihood of a developing an ischemic muscle contractility loss. SWE could possibly detect early shifts in the lower limb perfusion before NIRS-values are compromised. The early diagnosis is crucial, since lower limb ischemia in patients with VA-ECMO is associated with high mortality up to 70% [26]. Furthermore, every tenth patient requires surgical fasciotomy, which is an essential factor for the mortality rate [3].

A prospective study with children after surgery due to congenital heart disease explored the correlation between tissue oxygenation measured with NIRS and lactate levels. The authors showed weak correlations [27]. These previous findings support our hypothesis that oxygenation levels of the peripheral muscle tissue are difficult to determine, especially in an intensive care situation. Therefore, a decrease in muscle elasticity assessed by ultrasound shear wave elastography could help the detection of early limb ischemia. SWE examination can be performed in addition to the duplexsonography of the peripheral arteries; the latter one primary assessing severe blood flow obstructions of the macrovasculature. Our findings may have implications in the diagnostic management of the VA-ECMO therapy due to the risks of severe leg ischemia, which might initially be challenging to detect only with NIRS.

### Elasticity of the gastrocnemius muscle and peak systolic blood flow of the popliteal arteries during VA-ECMO

A regression analysis for a possible relationship between muscle elasticity measured by SWE during exercise and the peak systolic velocity of the popliteal artery is limited due to the small number of patients. However, the mean PSV on the cannulated side was reduced compared to the cannula-free side, even with a *p* value on the edge of significance. An exploratory study of nineteen VA-ECMO patients revealed a similar observation with PSV of the superficial femoral artery on the cannulated side being significantly reduced compared to the cannula-free sides [28]. Our finding supports the hypothesis that muscle elasticity and the haemodynamic state of the lower limbs affect each other. Muscle elasticity as a marker for the microcirculation deteriorates earlier and shows differences between the limbs before the peak systolic velocity between the sides is severely affected as a diagnostic tool for the microcirculation.

## Limitations

This study was designed as an explorative study. Limitations include the small cohort number and the required ultrasound training for examiners. Although the examination method is easy to perform and has previously demonstrated low inter-observer variability with unexperienced sonographers [18]. SWE cutoff values need to be established in larger controlled trials. This study is limited due to the absence of critical limb ischemia cases in these patients. In this way, we are unable to differentiate between clinically relevant and subclinical ischemia solely based on the muscle elasticity measured with SWE. Last, but not least, we did not perform follow-up examinations to obtain results in the course of VA-ECMO treatment. SWE cutoff values, especially for the state of critical limb ischemia, need to be established in larger controlled trials.

## Conclusion

Muscle ultrasound shear wave electrography (SWE) offers new insights in the dynamic changes of lower limb muscles and possible ischemia-related muscle function loss in patients with VA-ECMO. SWE is a rapid, non-invasive, bedside examination in critically ill patients. Combined with NIRS, it may provide additional information to help early identification of patients at risk for critical limb ischemia. Larger, controlled trials are necessary to provide reference values for muscle elasticity before and during limb ischemia in ICU patients.

## Data Availability

The data sets used and/or analysed during the current study are available from the corresponding author on reasonable request.

## Abbreviations

SWE: Shear wave elastography
VA-ECMO: Veno-arterial extracorporeal membrane oxygenation
NIRS: Near-infrared spectroscopy
DPC: Distal perfusion cannula
PSV: Peak systolic velocity
rSO_2_: Regional oxygen saturation
ROI: Region of interest
ICU: Intensive care unit
kPa: Kilopascal
CT-scan: Computed tomography-scan
PAD: Peripheral artery disease
